# Supplementary material for: Pyruvate Dehydrogenase Kinase 1 inhibition mediated oxidative phosphorylation enhancement in cartilage promotes osteoarthritis progression
Source: BMC Musculoskelet Disord. 2023 Jul 20;24:597. doi: 10.1186/s12891-023-06585-6 (PMC10357736; doi:10.1186/s12891-023-06585-6)
Supplement: Supplementary file 1 — Additional file 1. [file 12891_2023_6585_MOESM1_ESM.zip › BP.Gsea.1653623667859/GOBP_NADH_DEHYDROGENASE_COMPLEX_ASSEMBLY.html]

Details for gene set GOBP\_NADH\_DEHYDROGENASE\_COMPLEX\_ASSEMBLY[GSEA]

|  || Dataset | 55457\_collapsed\_to\_symbols.cls.cls #OA\_versus\_normal.cls.cls #OA\_versus\_normal\_repos |
| Phenotype | cls.cls#OA\_versus\_normal\_repos |
| Upregulated in class | OA |
| GeneSet | GOBP\_NADH\_DEHYDROGENASE\_COMPLEX\_ASSEMBLY |
| Enrichment Score (ES) | 0.69130486 |
| Normalized Enrichment Score (NES) | 2.701432 |
| Nominal p-value | 0.0 |
| FDR q-value | 0.0 |
| FWER p-Value | 0.0 |
Table: GSEA Results Summary

  

Fig 1: Enrichment plot: GOBP\_NADH\_DEHYDROGENASE\_COMPLEX\_ASSEMBLY      
 Profile of the Running ES Score & Positions of GeneSet Members on the Rank Ordered List

  

| SYMBOL | TITLE | RANK IN GENE LIST | RANK METRIC SCORE | RUNNING ES | CORE ENRICHMENT || 1 | NDUFA8 | NADH:ubiquinone oxidoreductase subunit A8 [Source:HGNC Symbol;Acc:HGNC:7692] | 67 | 0.961 | 0.0430 | Yes |
| 2 | TMEM186 | transmembrane protein 186 [Source:HGNC Symbol;Acc:HGNC:24530] | 128 | 0.876 | 0.0822 | Yes |
| 3 | NDUFS3 | NADH:ubiquinone oxidoreductase core subunit S3 [Source:HGNC Symbol;Acc:HGNC:7710] | 177 | 0.836 | 0.1204 | Yes |
| 4 | NDUFA1 | NADH:ubiquinone oxidoreductase subunit A1 [Source:HGNC Symbol;Acc:HGNC:7683] | 194 | 0.826 | 0.1605 | Yes |
| 5 | NDUFA13 | NADH:ubiquinone oxidoreductase subunit A13 [Source:HGNC Symbol;Acc:HGNC:17194] | 236 | 0.795 | 0.1972 | Yes |
| 6 | NDUFA10 | NADH:ubiquinone oxidoreductase subunit A10 [Source:HGNC Symbol;Acc:HGNC:7684] | 243 | 0.788 | 0.2362 | Yes |
| 7 | NDUFB6 | NADH:ubiquinone oxidoreductase subunit B6 [Source:HGNC Symbol;Acc:HGNC:7701] | 262 | 0.779 | 0.2738 | Yes |
| 8 | NDUFAF3 | NADH:ubiquinone oxidoreductase complex assembly factor 3 [Source:HGNC Symbol;Acc:HGNC:29918] | 285 | 0.761 | 0.3103 | Yes |
| 9 | TMEM126B | transmembrane protein 126B [Source:HGNC Symbol;Acc:HGNC:30883] | 295 | 0.753 | 0.3473 | Yes |
| 10 | COA1 | cytochrome c oxidase assembly factor 1 [Source:HGNC Symbol;Acc:HGNC:21868] | 426 | 0.695 | 0.3721 | Yes |
| 11 | NDUFB3 | NADH:ubiquinone oxidoreductase subunit B3 [Source:HGNC Symbol;Acc:HGNC:7698] | 483 | 0.667 | 0.4011 | Yes |
| 12 | NDUFS4 | NADH:ubiquinone oxidoreductase subunit S4 [Source:HGNC Symbol;Acc:HGNC:7711] | 531 | 0.647 | 0.4299 | Yes |
| 13 | NDUFA3 | NADH:ubiquinone oxidoreductase subunit A3 [Source:HGNC Symbol;Acc:HGNC:7686] | 550 | 0.641 | 0.4606 | Yes |
| 14 | NDUFA2 | NADH:ubiquinone oxidoreductase subunit A2 [Source:HGNC Symbol;Acc:HGNC:7685] | 878 | 0.549 | 0.4627 | Yes |
| 15 | NDUFB5 | NADH:ubiquinone oxidoreductase subunit B5 [Source:HGNC Symbol;Acc:HGNC:7700] | 902 | 0.542 | 0.4881 | Yes |
| 16 | NDUFC2 | NADH:ubiquinone oxidoreductase subunit C2 [Source:HGNC Symbol;Acc:HGNC:7706] | 929 | 0.532 | 0.5128 | Yes |
| 17 | NDUFA9 | NADH:ubiquinone oxidoreductase subunit A9 [Source:HGNC Symbol;Acc:HGNC:7693] | 943 | 0.529 | 0.5383 | Yes |
| 18 | NDUFB4 | NADH:ubiquinone oxidoreductase subunit B4 [Source:HGNC Symbol;Acc:HGNC:7699] | 968 | 0.525 | 0.5627 | Yes |
| 19 | NDUFB7 | NADH:ubiquinone oxidoreductase subunit B7 [Source:HGNC Symbol;Acc:HGNC:7702] | 1033 | 0.510 | 0.5833 | Yes |
| 20 | NDUFB1 | NADH:ubiquinone oxidoreductase subunit B1 [Source:HGNC Symbol;Acc:HGNC:7695] | 1055 | 0.506 | 0.6070 | Yes |
| 21 | NDUFS7 | NADH:ubiquinone oxidoreductase core subunit S7 [Source:HGNC Symbol;Acc:HGNC:7714] | 1071 | 0.501 | 0.6310 | Yes |
| 22 | NDUFC1 | NADH:ubiquinone oxidoreductase subunit C1 [Source:HGNC Symbol;Acc:HGNC:7705] | 1091 | 0.499 | 0.6545 | Yes |
| 23 | NDUFS8 | NADH:ubiquinone oxidoreductase core subunit S8 [Source:HGNC Symbol;Acc:HGNC:7715] | 1208 | 0.474 | 0.6693 | Yes |
| 24 | NDUFS1 | NADH:ubiquinone oxidoreductase core subunit S1 [Source:HGNC Symbol;Acc:HGNC:7707] | 1229 | 0.471 | 0.6913 | Yes |
| 25 | NDUFB11 | NADH:ubiquinone oxidoreductase subunit B11 [Source:HGNC Symbol;Acc:HGNC:20372] | 1603 | 0.413 | 0.6830 | No |
| 26 | NDUFS5 | NADH:ubiquinone oxidoreductase subunit S5 [Source:HGNC Symbol;Acc:HGNC:7712] | 2267 | 0.330 | 0.6480 | No |
| 27 | NDUFB2 | NADH:ubiquinone oxidoreductase subunit B2 [Source:HGNC Symbol;Acc:HGNC:7697] | 2460 | 0.307 | 0.6485 | No |
| 28 | NUBPL | nucleotide binding protein like [Source:HGNC Symbol;Acc:HGNC:20278] | 2569 | 0.295 | 0.6548 | No |
| 29 | NDUFB8 | NADH:ubiquinone oxidoreductase subunit B8 [Source:HGNC Symbol;Acc:HGNC:7703] | 2623 | 0.288 | 0.6651 | No |
| 30 | NDUFS2 | NADH:ubiquinone oxidoreductase core subunit S2 [Source:HGNC Symbol;Acc:HGNC:7708] | 3072 | 0.243 | 0.6425 | No |
| 31 | NDUFA6 | NADH:ubiquinone oxidoreductase subunit A6 [Source:HGNC Symbol;Acc:HGNC:7690] | 3834 | 0.173 | 0.5919 | No |
| 32 | NDUFAF1 | NADH:ubiquinone oxidoreductase complex assembly factor 1 [Source:HGNC Symbol;Acc:HGNC:18828] | 4388 | 0.122 | 0.5550 | No |
| 33 | NDUFAB1 | NADH:ubiquinone oxidoreductase subunit AB1 [Source:HGNC Symbol;Acc:HGNC:7694] | 4445 | 0.118 | 0.5566 | No |
| 34 | OXA1L | OXA1L mitochondrial inner membrane protein [Source:HGNC Symbol;Acc:HGNC:8526] | 5974 | -0.003 | 0.4379 | No |
| 35 | NDUFAF4 | NADH:ubiquinone oxidoreductase complex assembly factor 4 [Source:HGNC Symbol;Acc:HGNC:21034] | 6087 | -0.011 | 0.4298 | No |
| 36 | NDUFAF5 | NADH:ubiquinone oxidoreductase complex assembly factor 5 [Source:HGNC Symbol;Acc:HGNC:15899] | 6167 | -0.018 | 0.4245 | No |
| 37 | NDUFA5 | NADH:ubiquinone oxidoreductase subunit A5 [Source:HGNC Symbol;Acc:HGNC:7688] | 6789 | -0.062 | 0.3793 | No |
| 38 | BCS1L | "BCS1 homolog, ubiquinol-cytochrome c reductase complex chaperone [Source:HGNC Symbol;Acc:HGNC:1020]" | 7578 | -0.125 | 0.3243 | No |
| 39 | DMAC2 | distal membrane arm assembly component 2 [Source:HGNC Symbol;Acc:HGNC:25496] | 8347 | -0.182 | 0.2737 | No |
| 40 | NDUFAF7 | NADH:ubiquinone oxidoreductase complex assembly factor 7 [Source:HGNC Symbol;Acc:HGNC:28816] | 10077 | -0.328 | 0.1557 | No |
| 41 | TAFAZZIN | "tafazzin, phospholipid-lysophospholipid transacylase [Source:HGNC Symbol;Acc:HGNC:11577]" | 10429 | -0.364 | 0.1467 | No |
| 42 | MT-ND5 | mitochondrially encoded NADH:ubiquinone oxidoreductase core subunit 5 [Source:HGNC Symbol;Acc:HGNC:7461] | 12743 | -0.906 | 0.0122 | No |
Table: GSEA details [plain text format]

  

Fig 2: GOBP\_NADH\_DEHYDROGENASE\_COMPLEX\_ASSEMBLY      
 Blue-Pink O' Gram in the Space of the Analyzed GeneSet

  

Fig 3: GOBP\_NADH\_DEHYDROGENASE\_COMPLEX\_ASSEMBLY: Random ES distribution      
 Gene set null distribution of ES for **GOBP\_NADH\_DEHYDROGENASE\_COMPLEX\_ASSEMBLY**
